# Systemic Metal Ion Concentrations in Patients With Hip and Knee Megaprostheses: A Prospective Cohort Study

**DOI:** 10.1016/j.artd.2022.08.006

**Published:** 2022-10-21

**Authors:** Kiki Q. de Smidt, Geert Spierenburg, Richard E. Evenhuis, Sarah E. Bosma, Robert J.P. van der Wal, Demien Broekhuis, Michiel A.J. van de Sande

**Affiliations:** Department of Orthopaedics, Leiden University Medical Center, Leiden, The Netherlands

**Keywords:** Serum metal ion concentrations, (MoM) Megaprostheses, Aluminum, Cobalt, Chromium, Silver

## Abstract

**Background:**

Local tissue and serum metal ions have been shown to be elevated in some metal-on-metal and metal-on-polyethylene joint replacements. Local elevations have been linked to adverse local tissue reactions in some patients, and systemic elevation has been less commonly implicated cardiac and neurologic issues. Using a prospective study design, we aimed to identify the changes in serum metal ion levels after hip or knee megaprosthesis reconstruction. Furthermore, we will evaluate the occurrence of adverse effects and complications, possibly linked to metal ion elevation.

**Methods:**

Fourteen consecutive patients receiving a Modular Universal Tumor Revision System megaprosthesis were enrolled. Blood samples were collected preoperatively and postoperatively to determine the serum ion concentrations of aluminum, chromium, cobalt, and silver. To evaluate the safety of the megaprostheses and the subsequently possible related (elevated) serum metal ion concentrations, all adverse effects and complications were registered until last outpatient clinic visit at the time of this study.

**Results:**

Compared to the preoperative median serum concentrations, the postoperative median serum concentrations of chromium, silver, and cobalt increased 11-fold, 62-fold, and 64-fold, respectively. The median serum concentration of aluminum increased with 16%. Elevations were primarily noted in patients with knee prostheses. Eight patients had no adverse effects or complications during the period between preoperative and postoperative blood sampling. One adverse effect directly related to the serum metal ion concentrations, namely argyria, was observed.

**Conclusions:**

This study documents significantly elevated concentrations of the metal ions, but only one adverse effect directly related to the metal ion concentrations was observed. Future studies are needed to further assess the impact of elevated metal ion levels after megaprostheses, specifically knee implants, which are metal-on-metal.

## Introduction

Metal-on-metal (MoM) bearings have been developed to increase the mechanical durability of the prosthesis compared to the conventional metal-on-polyethylene (MoP) bearings [1,2]. MoM hinges are used, among other things, in megaprostheses about the knee to reconstruct osseous defects after tumor resections as they have clear benefits preserving function and quality of life [3,4].

Several studies documented elevated serum metal ion levels and (local) adverse reactions to metal debris in patients with MoM prostheses, such as metallosis, osteolysis, and pseudotumor formation [[5], [6], [7], [8], [9], [10]]. Furthermore, revision surgery and a higher mortality have been described as well [2,11]. Mechanisms contributing to metal ions release include MoM articulation, corrosion of nonarticulating surfaces, fretting of modular junctions, and abrasive wear of soft tissues [5]. These concerns have led to questions about the use of MoM hinged megaprostheses about the knee and general megaprostheses containing large surfaces of metal.

A limited number of studies intended to describe and quantify the systemic metal ion concentrations and the effects of several metal ions from corresponding alloying elements in megaprostheses [1,5,10,12]. Metals often used in alloying elements of megaprostheses are aluminum (Al), chromium (Cr), cobalt (Co), molybdenum (Mo), silver (Ag), titanium (Ti), and vanadium (V) [13]. Among these metals, silver is an encouraging option to reduce the rate of infection because of its relatively low toxicity and high level of antimicrobial activity [[14], [15], [16]]. However, silver ions are reported to cause adverse effects, such as argyria, neuropathy, hepatic failure, and renal failure [17]. In the case of cobalt, mainly cardiovascular and neurologic adverse effects are reported [18]. For other metal ions, such as aluminum and titanium, little data are published about systemic concentrations and their possible adverse effects.

This prospective study documents the change in serum metal ion levels preoperatively and postoperatively in patients with megaprostheses. As the global utilization of megaprostheses is usually confined to patients suffering from malignant bone tumors, data on metal ion dissemination up till now remain limited. We aimed to investigate several serum metal ion concentrations from corresponding alloying elements in patients with hip and knee megaprostheses, before and after surgery. Furthermore, we looked at factors correlating to metal ion release and the occurrence of adverse effects and complications.

## Material and methods

### Design and patients

**Figure 1:**
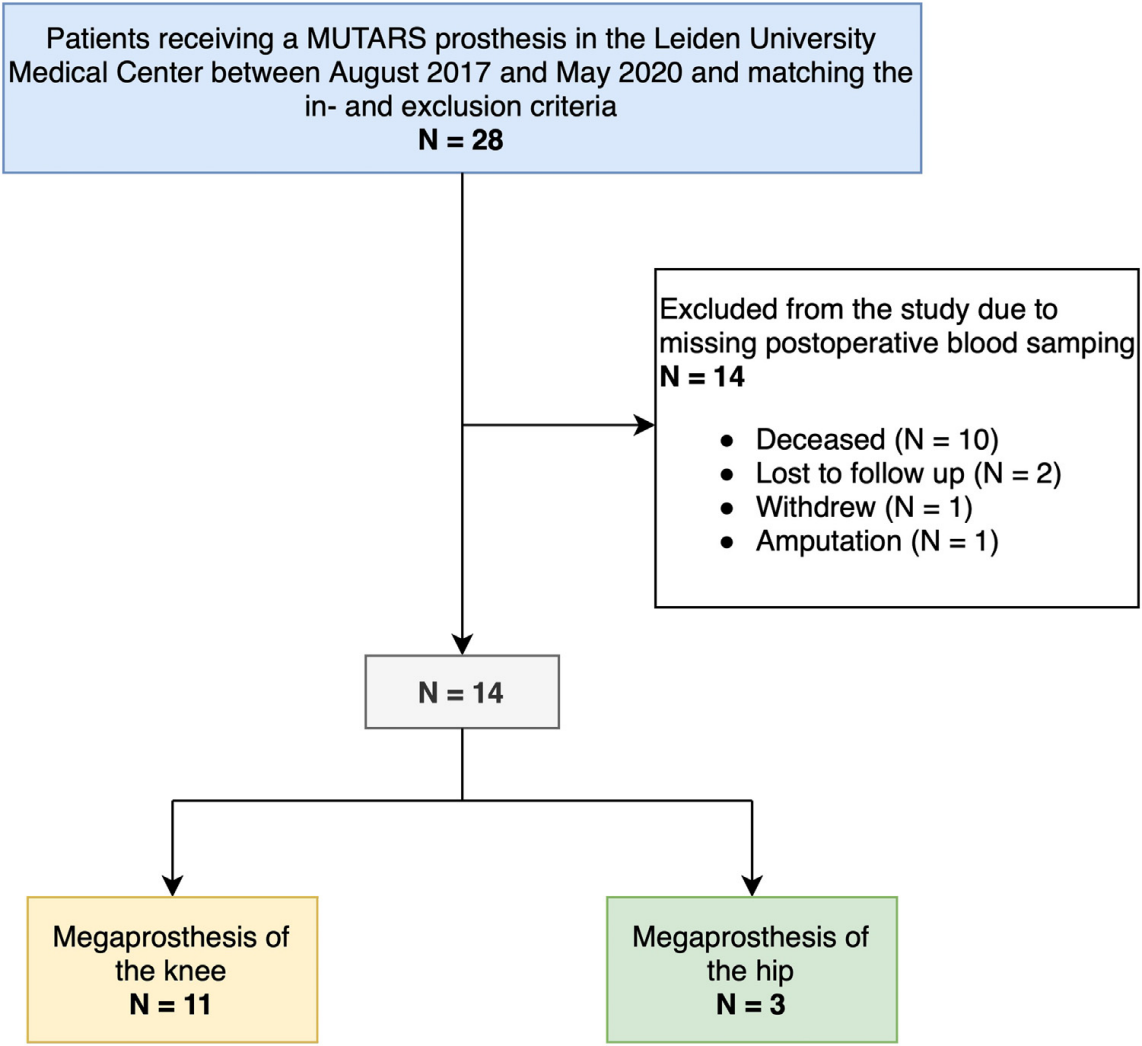
Inclusion flowchart.

This is a prospective cohort study describing metal ion concentrations in blood serum before and after megaprostheses implantation in 14 consecutive patients. Secondary, adverse effects and complications were recorded. Between August 2017 and May 2020, all consecutive patients in the Leiden University Medical Center aged ≥18 years and receiving an endoprosthetic reconstruction using Modular Universal Tumor Revision System (MUTARS®; Implantcast GmbH, Buxtehude, Germany) hinge were considered for the study. Exclusion criteria were anamnestic use of metal-containing nutritional supplements or medications, contact with metal ions in the work environment, an estimated glomerular filtration rate <60, any other in situ prosthesis containing metal, and revision surgery of any metallic prosthesis. A total of 28 patients receiving a megaprosthesis within the study period were included. Before the postoperative blood sampling, 10 patients had died due to progression of the sarcoma, 2 patients were lost to follow-up, 1 patient underwent amputation after tumor recurrence before postoperative blood sampling, and 1 patient had withdrawn from the study (Fig. 1).

**Figure 2:**
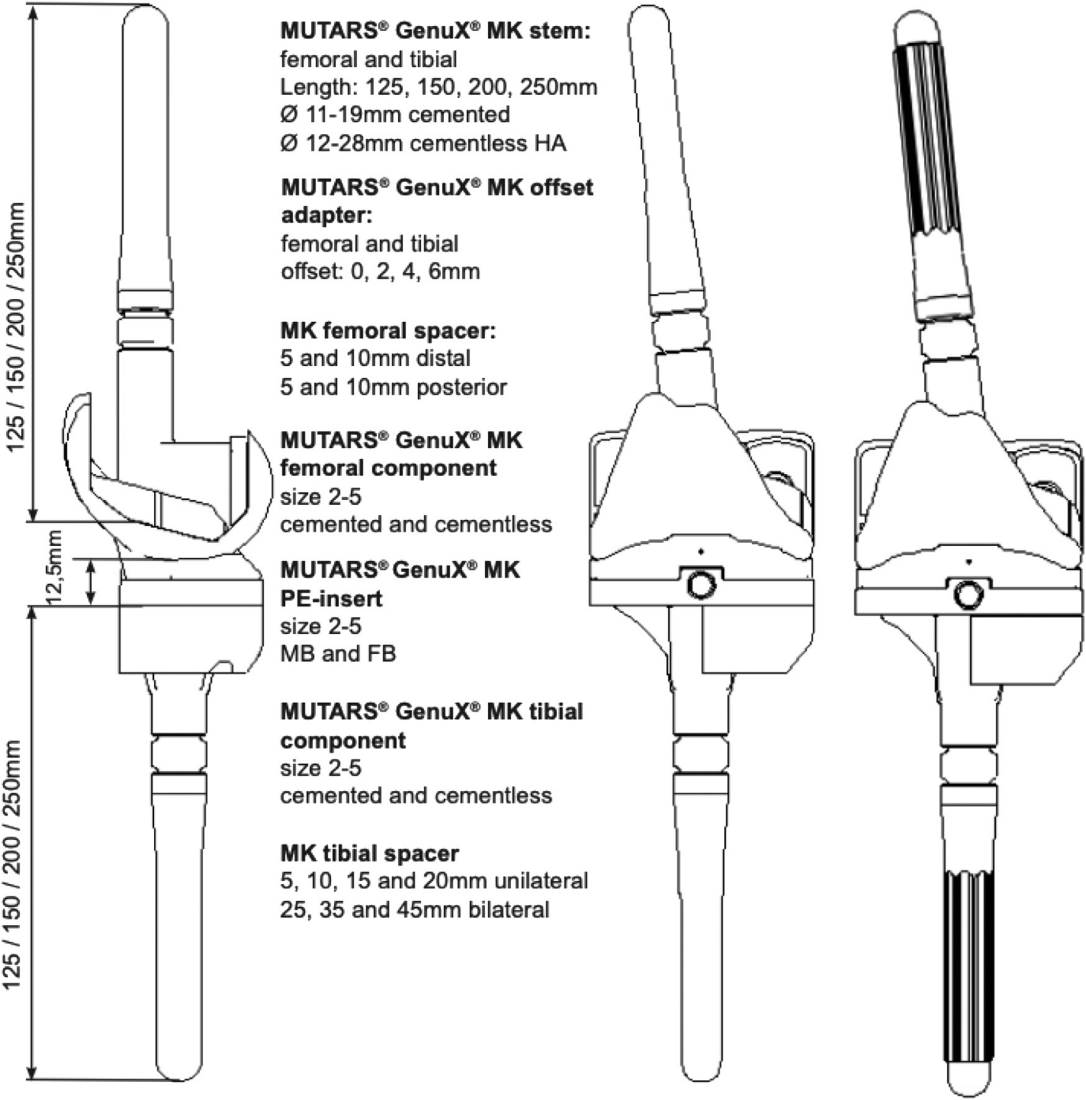
An image demonstrating the MUTARS® total knee arthroplasty (TKA) GenuX® MK System. MUTARS®, Modular Universal Tumor Revision System.

Reproduced with permission from implantcast GmbH.

Eleven patients requiring a knee megaprosthesis, received the total knee arthroplasty (TKA) GenuX® MK System (Implantcast GmbH, Buxtehude, Germany) (Fig. 2) [19]. The coupling mechanism and tibial and femoral components of the MUTARS® GenuX® MK are made of a CoCrMo alloy according to the International Standard Organization (ISO) 5832/12 [19]. The offset adapter and femoral and tibial spacers used in these prostheses are made of a TiAl6V4 alloy (ISO 5832/3). In this cohort, only cementless stems made of a TiAl6V4 alloy were used (ISO 5832/3).

**Figure 3:**
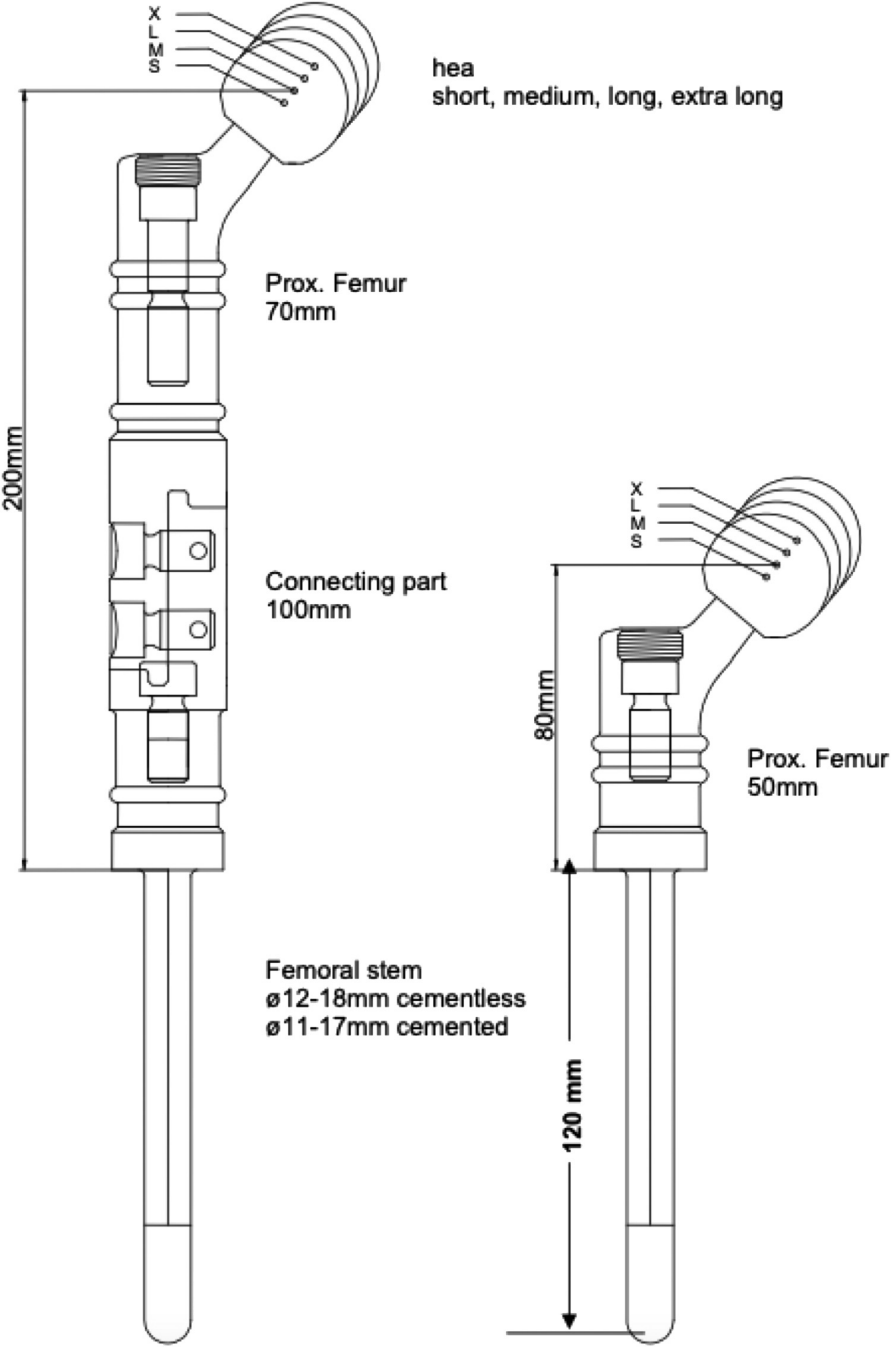
An image demonstrating the MUTARS® proximal femur. MUTARS®, Modular Universal Tumor Revision System.

Reproduced with permission from implantcast GmbH.

Three patients requiring a hip megaprosthesis received the MUTARS® proximal femur (Implantcast GmbH, Buxtehude, Germany) (Fig. 3) [20]. According to the ISO, the MUTARS® proximal femur, the extension piece, and the connecting part are made of a TiAl6V4 alloy, as well as cementless stems (ISO 5832/3) [20]. All stems were uncemented. Furthermore, 1 patient received both a MUTARS® LUMiC and a MUTARS® proximal femur of the hip. In this cohort, all femoral heads were bipolar heads and consisted of CoCrMo alloy and ultra-high-molecular-weight polyethylene. Therefore, these proximal femur megaprostheses are MoP prostheses, unlike the MoM knee megaprosthesis used in this study.

All prostheses used in this study thus contained components made of both CoCrMo and TiAl6V4 alloys and all prostheses, except 1, were silver coated. No implant loosening was observed.

### Outcome measures

The primary outcomes were changes in serum concentrations of Co, Cr, Al, and Ag following surgery with a MUTARS® megaprosthesis. Blood samples of all patients were collected preoperatively on the day of the surgery and postoperatively during outpatient clinic visits. The blood samples were analyzed with inductively coupled plasma mass spectrometry (ICP-MS 350D) (PerkinElmer, Waltham, Massachusetts, USA).

All serum metal ion concentrations were expressed as nmol/l or μmol/l. To convert μmol/l to part per billion (ppb), the concentration of the metal ion (in μmol/l) was multiplied by the molar mass [21]. Reference values of the laboratory for the metal ion concentrations in serum were 0.32-1.74 ppb for Cr, <9.98 ppb for Al, <5.00 ppb for Ag, and 0.30-1.02 ppb for Co.

The secondary outcome was the safety of (MoM) MUTARS® megaprostheses by evaluating all adverse effects and (mechanical) complications of the prosthesis, including reported local and systemic responses following MoM hip implantations such as pseudotumor formation, argyria, blindness, and cardiopathy, until last outpatient clinic at the time of this study [22]. Radiographical images were assessed for mechanical failure, osteolysis, and implant loosening. Serum tests were analyzed to assess possible systemic adverse effects. The complications were recorded and, if possible, classified according to the Henderson classification, version 2.0. All complications were eventually divided into 2 groups: (1) adverse effects and complications from the implantation of the prosthesis until the postoperative blood sampling and (2) adverse effects and complications from the postoperative blood sampling until last outpatient clinic [23].

### Statistics

Means and standard deviations or 95% confidence intervals were used for normally distributed descriptive variables. Medians and interquartile ranges (IQRs) or ranges were used for skewed data. The Wilcoxon signed-rank test was used to compare the median preoperative and postoperative metal ion levels. SPSS (IBM SPSS Statistics 25.0; IBM Corp, Armonk, NY, USA) was used for the analyses.

### Ethics, registration, funding, and potential conflicts of interest

Approval for the present study was waived by the Medical Research Ethics Committee Leiden-Den Haag-Delft, the Netherlands (P17.058) and informed consent was acquired from all patients prior to inclusion. The study was conducted according to the Declaration of Helsinki (October 2013) and in accordance with the Medical Research Involving Human Subjects Act. No funding was obtained for the present study, and none of the authors have reported a potential conflict of interest.

## Results

A total of 14 patients with a MUTARS® tumor prosthesis participated in the follow-up (Fig. 1).

The median age of all patients was 54.5 years (IQR 40.5-68.0), 7 patients (50%) were men, and the median body mass index was 25.2 kg/m^2^ (IQR 23.5-28.8) (Table 1). The median time to postoperative blood sampling was 17.5 months (IQR 15.5-27.0). The primary tumor was located in the proximal tibia in 4 patients (29%), in the distal femur in 7 patients (50%), and in the proximal femur in 3 patients (21%). Four patients (29%) were diagnosed with osteosarcoma, 2 patients (14%) with giant cell tumor, 5 patients (36%) with chondrosarcoma, 1 patient with leiomyosarcoma (7%), 1 patient (7%) with undifferentiated pleomorphic sarcoma, and 1 patient (7%) was diagnosed with diffuse large B cell lymphoma. Two patients (14%) underwent preoperative chemotherapy, 3 patients (21%) underwent postoperative chemotherapy, and 1 patient (7%) underwent postoperative radiotherapy. Ninety-three percent of the patients had a silver coating on the body of the prosthesis. In Table 2, the lengths of the components of the protheses are reported for all patients.

**Table 1:** Patient characteristics.

Variable	Total	Megaprosthesis in the knee	Megaprosthesis in the hip
	N = 14	N = 11	N = 3
Male, N (%)	7 (50)	5 (45)	2 (67)
Age at surgery, median y (IQR)	54.5 (40.5-68.0)	54.0 (43.0-71.0)	55.0 (31.0-)
Follow-up of the blood sampling, median mo (IQR)	17.5 (15.5-27.0)	18.0 (16.0-30.0)	17.0 (13.0-)
BMI, median kg/m^2^ (IQR)	25.2 (23.5-28.8)	25.4 (23.9-30.1)	24.7 (19.9-)
Side, N (%)			
Left	11 (79)	8 (73)	3 (100)
Right	3 (21)	3 (27)	0 (0)
Location tumor, N (%)			
Proximal tibia	4 (29)	4 (36)	0 (0)
Distal femur	7 (50)	7 (64)	0 (0)
Proximal femur	3 (21)	0 (0)	3 (100)
Type tumor, N (%)			
Osteosarcoma	4 (29)	4 (36)	0 (0)
GCTB	2 (14)	2 (18)	0 (0)
Chondrosarcoma	5 (36)	2 (18)	3 (100)
Leiomyosarcoma	1 (7)	1 (9)	0 (0)
UPS	1 (7)	1 (9)	0 (0)
DLBCL	1 (7)	1 (9)	0 (0)
Preoperative radiology, N (%)	0 (0)	0 (0)	0 (0)
Postoperative radiology, N (%)	1 (7)	1 (9)	0 (0)
Preoperative chemotherapy, N (%)	2 (14)	2 (18)	0 (0)
Postoperative chemotherapy, N (%)	3 (21)	3 (27)	0 (0)
Silver coating, N (%)	13 (93)	10 (91)	3 (100)

BMI, body mass index; DLBCL, diffuse large B cell lymphoma; GCTB, giant cell tumor of the bone; IQR, interquartile range; UPS, undifferentiated pleomorphic sarcoma.

**Table 2:** Data of all patients with respect to the tumor, the prosthesis, complications, and adverse effects.

Patient. No.	Site	Time to the blood sampling (mo)	Silver coating	Type of tumor	Length of the femur body (mm)	Length of the femur stem (mm)	Length of the tibia body (mm)	Length of the tibia stem (m)	Complications and adverse effects until postoperative blood sampling	Complications and adverse effects after postoperative blood sampling until last outpatient clinic
1	Distal femur, L	14	Yes	Osteosarcoma	297	143		188		Recurrence of the tumor (*Henderson type 5 failure*)
2	Proximal tibia, R	23	Yes	Osteosarcoma		187	184	143	Peroneus neuropraxia	
3	Distal femur, L	18	Yes	GCTB	192	144		146		
4	Distal femur, R	26	Yes	Osteosarcoma	191	148		180		
5	Proximal tibia, L	40	Yes	Chondrosarcoma		181	176	139		
6	Distal femur, L	30		Leiomyosarcoma	135	125		154		
7	Distal femur, L	16	Yes	Chondrosarcoma	147	210		175	Prosthesis infection → DAIR (*Henderson type 4A failure*)	
8	Proximal tibia, R	17	No	GCTB		191	162	142	Drop foot and wound dehiscence → VAC (*Henderson type 1B failure*)	
9	Distal femur, L	13	Yes	Osteosarcoma	166	147		145		
10	Proximal tibia, L	17	Yes	UPS		235	128	137		Amputation after tumor recurrence (*Henderson type 5 failure*)
11	Proximal femur, L	21	Yes	Chondrosarcoma	158	149			Persistent pain, tumor recurrence (*Henderson type 5 failure*)	
12	Proximal femur, L	17	Yes	Chondrosarcoma	182	145			Argyria, DVT	
13	Proximal femur, L	13	Yes	Chondrosarcoma	157^a^	230^a^				
14	Distal femur, L	49	Yes	DLBCL	277	149		134	Instability, car accident → revision hinge and liner (*Henderson type 3A failure*)	

DAIR, debridement; antibiotics and implant retention; DLBCL, diffuse large B cell lymphoma; DVT, deep vein thrombosis; GCTB, giant cell tumor of the bone; UPS, undifferentiated pleomorphic sarcoma; VAC, vacuum-assisted closure.

a MUTARS® proximal femur + MUTARS® LUMiC.

### Metal ion concentrations

**Figure 4:**
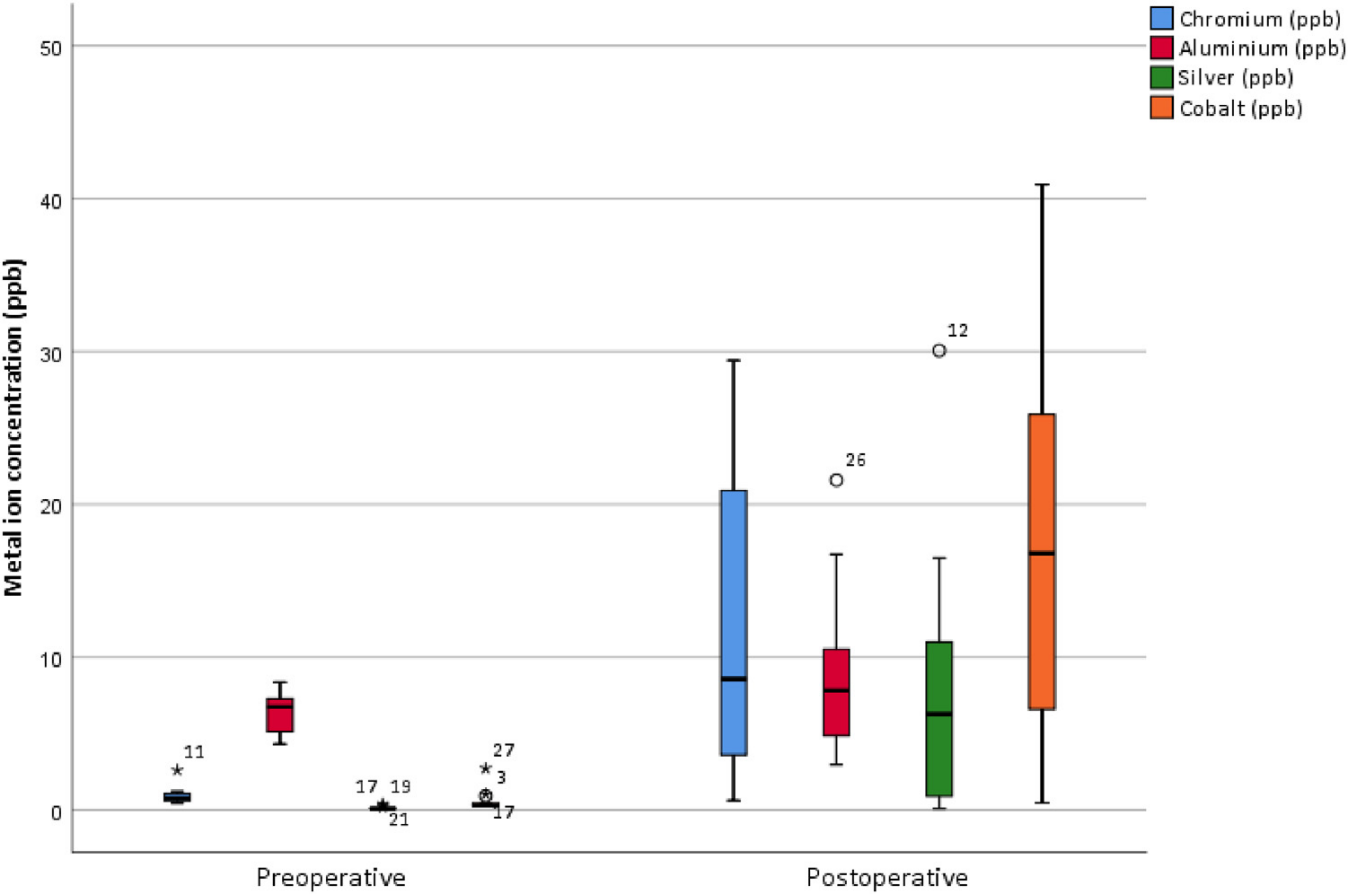
Boxplot showing preoperative and postoperative serum levels of chromium, aluminum, silver, and cobalt in parts per billion (ppb). The median follow-up was 17.5 months.

**Figure 5:**
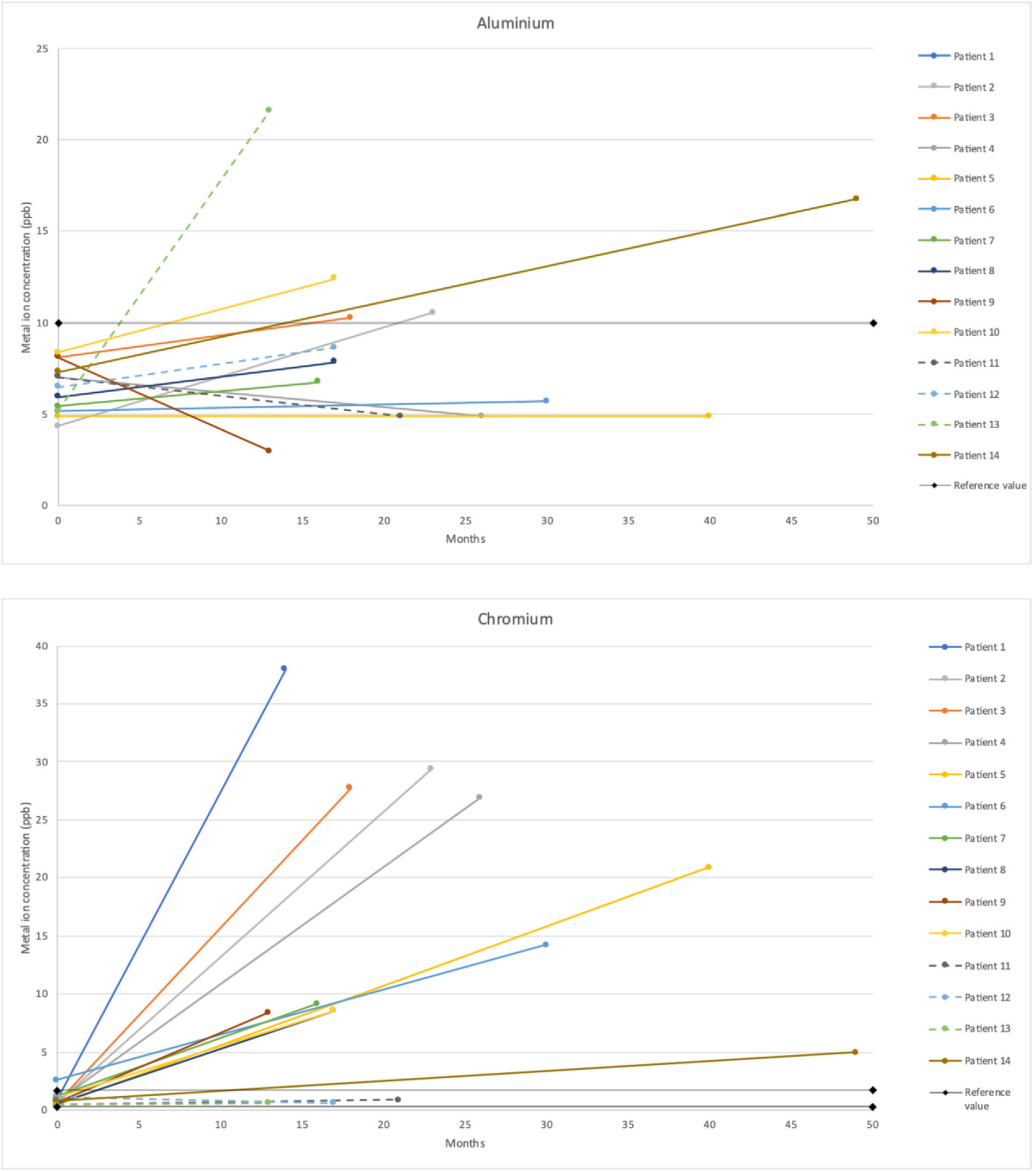

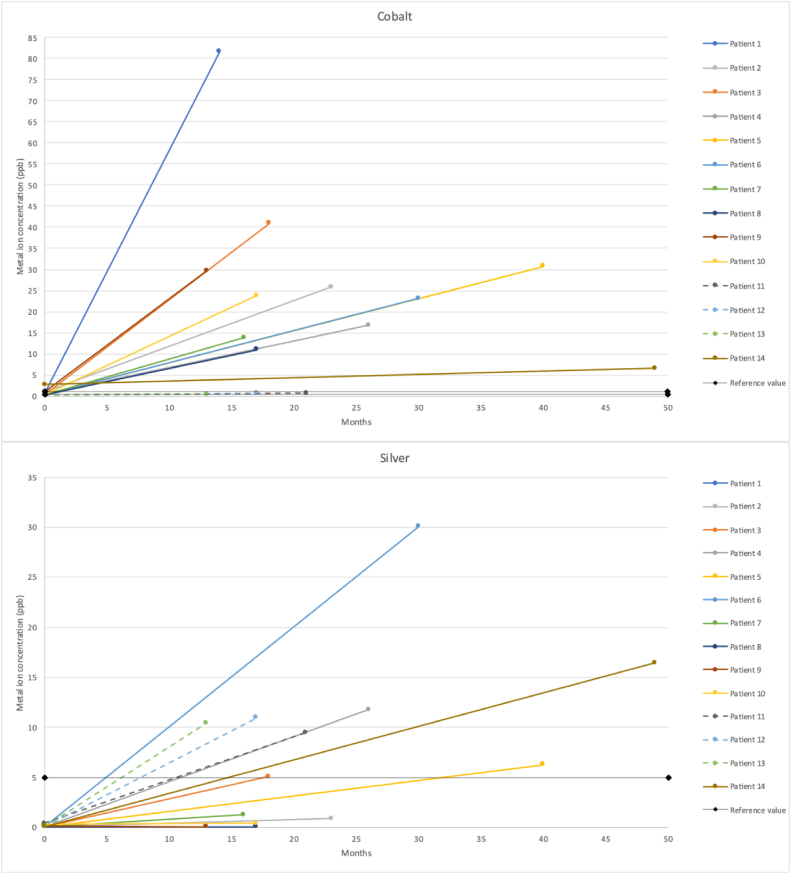
Individual courses of serum ion concentrations of chromium, aluminum, silver, and cobalt, in parts per billion (ppb). The dotted lines represent the patients with a megaprosthesis of the hip. The continuous lines represent the patients with a megaprosthesis of the knee. The reference values are the following: chromium 0.32-1.74 ppb, aluminum <9.98 ppb, silver <5.00 ppb, and cobalt 0.30-1.02 ppb.

The preoperative median concentrations of Cr, Al, Ag, and Co in the serum of patients with a MUTARS® megaprosthesis were 0.78 (IQR 0.59-1.01), 6.75 (IQR 5.13-7.49), 0.10 (IQR 0.10-0.13), and 0.31 (IQR 0.27-0.56), respectively (Table 3a, Fig. 4). Thirteen patients (93%) had a preoperative serum ion concentration within the reference values for Cr, 14 patients (100%) for Al, 14 patients (100%) for Ag, and 6 patients (43%) for Co (Fig. 5). The postoperative median concentrations of Cr, Al, Ag, and Co in the serum were 8.87 (IQR 2.93-27.12), 7.82 (IQR 4.86-11.47), 6.29 (IQR 0.65-11.39), and 19.97 (IQR 5.12-29.98), respectively (Table 3a, Fig. 4). Three patients (21%) had a postoperative serum ion concentration within the reference values for Cr. All of them had a megaprosthesis of the hip. Eight patients (57%) had a serum ion concentration within the reference values for Al, although the concentration of 1 patient was missing. For Ag, 5 patients (36%) were within the reference values. The Ag concentration of 1 patient was missing. Three patients (21%), of whom all had a megaprosthesis of the hip, were within the reference values for Co (Fig. 5). Compared to the upper limit of the reference values, the postoperative median serum concentrations of Cr and Co, were 5-fold and 20-fold, respectively. The postoperative median serum concentration of Ag was 26% higher than the upper limit of the reference value. The postoperative median serum concentration of Al stayed within the reference values. Compared to the preoperative median serum concentrations, the postoperative median serum concentrations of Cr, Ag, and Co increased 11-fold, 63-fold, and 64-fold, respectively. The median serum concentration of Al increased with 16%.

**Table 3a:** Metal ion concentrations total.

Serum metal ion levels		Postoperative compared to preoperative	Wilcoxon signed rank-test (effect size, r)
Serum chromium ion level, median ppb (IQR)			
Preoperative	0.78 (0.59-1.01)	x 11	*P* = .002 (−0.59)
Postoperative	8.87 (2.93-27.12)		
Serum aluminum ion level, median ppb (IQR)			
Preoperative	6.75 (5.13-7.49)	+16%	*P* = .157 (−0.28)
Postoperative	7.82 (4.86-11.47)		
Serum silver ion level, median ppb (IQR)			
Preoperative	0.10 (0.10-0.13)	x 63	*P* = .003 (−0.58)
Postoperative	6.29 (0.65-11.39)		
Serum cobalt ion level, median ppb (IQR)			
Preoperative	0.31 (0.27-0.56)	x 64	*P* = .001 (−0.62)
Postoperative	19.97 (5.12-29.98)		

IQR, interquartile range; Ppb, parts per billion.

**Table 3b:** Metal ion concentrations in patients with a megaprosthesis in the knee.

Serum metal ion levels		Postoperative compared to preoperative	Wilcoxon signed-rank test (effect size, r)
Serum chromium ion level, median ppb (IQR)			
Preoperative	0.82 (0.66-1.08)	x 17	*P* = .003 (−0.63)
Postoperative	14.23 (8.44-27.73)		
Serum aluminum ion level, median ppb (IQR)			
Preoperative	7.02 (5.13-8.09)	+4%	*P* = .192 (−0.29)
Postoperative	7.29 (4.86-10.99)		
Serum silver ion level, median ppb (IQR)			
Preoperative	0.10 (0.10-0.10)	x 31	*P* = .011 (−0.57)
Postoperative	3.19 (0.33-12.96)		
Serum cobalt ion level, median ppb (IQR)			
Preoperative	0.34 (0.27-0.92)	x 70	*P* = .003 (−0.63)
Postoperative	23.79 (13.84-30.70)		

IQR, interquartile range; Ppb, parts per billion.

### Complications and adverse effects

A total of 8 patients (57%) had complications or adverse effects during the total follow-up. One patient (7%) developed argyria, which was directly related to the elevated serum concentration of metal ions (Table 2). The serum Ag concentration of this patient was 10.99 ppb. One patient (7%) had a complication according to Henderson type 1B (wound dehiscence), 1 patient (7%) according to Henderson type 3A after a car accident (revision of the hinge and the liner), 1 patient (7%) had a complication according to Henderson type 4A (periprosthetic joint infection requiring debridement, antibiotics, irrigation, and retention), and 3 patients (21%) according to Henderson type 5 (tumor recurrence). None of these patients had outlying postoperative metal ion concentrations. Eight patients (57%) had neither any adverse effects nor complications during the period between the preoperative and postoperative blood sampling.

**Table 3c:** Metal ion concentrations in patients with a megaprosthesis in the hip.

Serum metal ion levels		Postoperative compared to preoperative	Wilcoxon signed-rank test (effect size, r)
Serum chromium ion level, median ppb (range)			
Preoperative	0.57 (0.45-1.17)	+12%	*P* = 1.000 (0.00)
Postoperative	0.64 (0.62-0.88)		
Serum aluminum ion level, median ppb (range)			
Preoperative	6.48 (5.13-7.02)	+33%	*P* = .593 (−0.24)
Postoperative	8.63 (4.86-21.59)		
Serum silver ion level, median ppb (range)			
Preoperative	0.10 (0.10-0.40)	x 104	*P* = .109 (−0.65)
Postoperative	10.46 (9.49-10.99)		
Serum cobalt ion level, median ppb (range)			
Preoperative	0.32 (0.24-0.32)	+65%	*P* = .109 (−0.65)
Postoperative	0.53 (0.47-0.68)		

Ppb, parts per billion.

## Discussion

The present study assessed the change in serum metal ion concentrations preoperatively and postoperatively and the adverse effects and complications in patients receiving a MUTARS® megaprosthesis. Compared to the preoperative median serum concentrations, the postoperative median serum concentrations of Cr, Ag, and Co increased 11-fold, 63-fold, and 64-fold, respectively. The median serum concentration of Al increased with 16%. Postoperatively, only the median serum ion concentration of Al stayed within the reference values. Eight patients (57%) had no adverse effects or complication during the period between the preoperative and postoperative blood sampling. One adverse effect, which was supposed to be directly related to the serum metal ion concentrations, was observed.

To our best knowledge, limited data have been published concerning serum metal ion levels after implantation of a knee megaprosthesis. Friesenbichler et al. (2014) [10] reported serum metal ion concentrations of Co, Cr, and Mo in pediatric patients who underwent TKA using megaprostheses. They found a mean Co and Cr concentration of 7.50 ppb (range 0-47 ppb) and 2.98 ppb (range 0.12-24.90 ppb), respectively. In 2017, Laitinen et al. [1] compared MoM MUTARS® TKA to MoP MUTARS® TKA. They found higher whole blood concentrations of Co and Cr in patients with an MoM prosthesis (Co 17.80 ppb vs 1.7 ppb, Cr 6.6 ppb vs 1.4 ppb). In comparison, serum metal ion concentrations are generally higher than whole blood metal ion concentrations [24]. Klasan et al. (2019) [12] measured the postoperative Co and Cr serum concentrations in patients receiving the same megaprosthesis in the knee as in this study, the GenuX® MK System. The median postoperative serum Cr level was 6.3 ppb (range 0.6-31.9 ppb) and the median serum Co level was 10.5 ppb (range 1.0-47.5 ppb) (Supplementary Table 1). Compared to previous studies, our postoperative median serum Co and Cr concentrations (19.97 and 8.87 ppb) are relatively high. Although the median follow-up of all studies differs, which could contribute to the difference in Co and Cr ion concentrations between the studies, Laitinen et al. showed that the median Co concentration increased between 1 and 2 years postoperatively. This may suggest that postoperative serum Co concentration measured in our study might even increase when the follow-up would be extended. However, none of these studies included the preoperative serum metal ion concentrations. Therefore, the absolute differences could not be compared.

In this relatively short-term study, metal ion release of especially Co and Cr from the MoM knee megaprostheses appeared to be substantially higher than released from the hip megaprostheses. This can be attributed due to fact that the articulating part of these prostheses were made of both ultra-high-molecular-weight polyethylene and a CoCrMo alloy and not MoM. Contrarily, the hinge in the MUTARS® megaprostheses of the knee is exclusively made of a CoCrMo alloy, which we expect as the main cause for elevated levels of Co and Cr. As inductively coupled plasma mass spectrometry is not able to discriminate between various atomic (ie, ionized/nonionized/nanoparticulate, etc) forms of metals, it was not feasible to differentiate for possible other mechanisms contributing to the (differences in) metal ion release.

Only 1 adverse effect directly related to elevated metal ion concentrations was observed, namely argyria. This patient had a postoperative serum Ag concentration of 10.99 ppb. However, other patients with a postoperative serum Ag concentration above this value (up to 30.06 ppb) did not show any signs of argyria. Moreover, the patients with the highest postoperative serum Ag concentrations did not have the largest Ag-coated surfaces. Glehr et al. (2013) [17] also concluded that the length of the Ag-coated implant did not influence the development of local argyria. In this study, 23% of the patients developed local argyria. The levels of Ag in the blood of patients with and without argyria were comparable. Although no other cases of adverse local tissue reaction were found in our patients, pseudotumor formation in patients with MoM THAs and TKAs have been documented in other studies and case reports [6,25,26]. Sutphen et al. (2015) [6] showed that 68.8% of their patients developed a pseudotumor, mostly asymptomatic. They found no difference with elevated serum metal ions and prevalence of pseudotumor. However, in the study of McGrory et al. (2016) [27], the relationship between serum and intra-articular Co and Cr were examined, showing intra-articular levels 100 times higher than serum levels. This may explain adverse effects in patients with relatively low metal ion serum values.

One limitation of this study is the absence of uniform postoperative follow-up moments for all patients. Subsequently, the time between the preoperative and postoperative blood sampling is not equal and the postoperative serum metal ion concentrations are not directly comparable. Another limitation is the small study population, predominantly caused by death of patients before the postoperative blood sampling. Therefore, the statistical comparisons should be made with caution. The coronavirus disease 2019 pandemic and the associated scaling down of regular care contributed to both limitations. In addition, the follow-up is relatively short, and thus, the total course of the serum metal ion concentrations in this study could not be followed up extensively. A long-term prospective study with fixed follow-up moments would result in better understanding of this course.

To date, metal ion monitoring is only recommended for patients with MoM hip prostheses [[28], [29]]. On MoM knee prostheses, and especially megaprostheses, as in this study, no guidelines or recommendations have yet been published. Since the patient group receiving megaprostheses often undergo (neo-)adjuvant chemotherapy and the adverse effects related to elevated metal ion concentrations partly overlap with the adverse effects related to chemotherapy, this situation of MoM hip prostheses and (MoM) megaprostheses is not comparable. Therefore, the recommendations regarding metal ion testing in patients with MoM hip prostheses cannot directly be applied for megaprostheses, and more research with larger patient groups and longer follow-up on metal ion release following (knee) megaprostheses should be conducted in order to create guidelines for metal ion testing in patients with a (MoM) megaprosthesis.

## Conclusions

To conclude, this study documents elevated serum metal ion concentrations in especially Ag, Cr, and Co compared to the preoperative concentrations in patients with a megaprosthesis. The metal ions released by the megaprostheses seemed to be well tolerated in most patients; the most pronounced adverse effect in this case series was 1 case of argyria out of 11 patients that had a silver-coated prosthesis. Therefore, long-term prospective studies in larger patient groups are needed to further assess the relation between (MoM) megaprostheses and local and systemic metal ions.

## Conflicts of interest

The authors declare there are no conflicts of interest.

For full disclosure statements refer to https://doi.org/10.1016/j.artd.2022.08.006.

## Figures and Tables

**Supplementary Table 1:** Present study compared to prior studies.

Variable	Friesenbichler et al. (2014) [10]	Laitinen et al. (2017) [1]	Klasan et al. (2019) [12]	Present study
Co (ppb)	7.50 (mean, serum)	17.80 (mean, whole blood)	10.50 (median, serum)	19.97 ppb (median, serum)
Cr (ppb)	2.98 (mean, serum)	6.60 (mean, whole blood)	6.30 (median, serum)	8.87 ppb (median, serum)
Megaprosthesis	LPS (LPS/M.B.T.; DePuy, Warsaw, IN, USA)	MoM MUTARS® hinge TKA (MUTARS®; Implantcast, Buxtehude, Germany)	MoM MUTARS® GenuX® MK System (MUTARS®; Implantcast, Buxtehude, Germany)	MoM MUTARS® GenuX® MK + MUTARS® proximal femur (MUTARS®; Implantcast, Buxtehude, Germany)
FU (months)	35 (mean)	36 (mean)	28 (median)	17.5 (median)

## Appendix A Supplementary data

Conflict of Interest Statement for Broekhuis

Conflict of Interest Statement for Spierenburg

Conflict of Interest Statement for de Smidt

Conflict of Interest Statement for van de Sande

Conflict of Interest Statement for Evenhuis

Conflict of Interest Statement for Bosma

Conflict of Interest Statement for van der Wal
