# Supplementary material for: Systemic Metal Ion Concentrations in Patients With Hip and Knee Megaprostheses: A Prospective Cohort Study
Source: Arthroplast Today. 2022 Oct 21;18:191–201.e1. doi: 10.1016/j.artd.2022.08.006 (PMC9764025; doi:10.1016/j.artd.2022.08.006)
Supplement: Conflict of Interest Statement for van de Sande [file mmc4.docx]

# INDIVIDUAL CONFLICT OF INTEREST STATEMENT

***American Association of Hip and Knee Surgeons***

(Adopted from the American Academy of Orthopaedic Surgeons disclosure statement)

The following form **must be filled out completely and submitted by each author (example, 6 authors, 6 forms).**

**All items require a response. If there is no relevant disclosure for a given item, enter "*None*.”**

**Manuscript Title**

1. **NO** Royalties from a company or supplier

2. Speakers bureau/paid presentations for a company or supplier **NONE**

3A. Paid employee for a company or supplier **NONE**

3B. Paid consultant for a company or supplier **NONE**

3C. Unpaid consultants for a company or supplier IMPLANTCAST GMBH

4. Stock or stock options in a company or supplier **NONE**

5. Research support from a company or supplier as a Principal Investigator Research grant to department of orthopedics LUMC by IMPLANTCAST GMBH, Carbofix, Daiichy Sankyo, Livemax

6. Other financial or material support from a company or supplier **NONE**

7. Royalties, financial or material support from publishers **NONE**

8. Medical/Orthopaedic publications editorial/governing board **NONE**

9. Board member/committee appointments for a society **EMSOS, EE, WEBOT**

**Each author must sign AND print or type his/her name, date and submit a separate form**

In addition, one BLINDED Conflict of Interest form (no author names used) should be submitted per manuscript with all author disclosures.

Author Name Maj van de Sande Author Signature Date 4-1-2022
